# Exceptional use: examining methyl bromide applications in California 2016–2022

**DOI:** 10.1088/2515-7620/ae3227

**Published:** 2026-01-12

**Authors:** Yoshira Ornelas Van Horne, Jill E Johnston

**Affiliations:** 1 Department of Environmental Health Sciences, Fielding School of Public Health, University of California Los Angeles, Los Angeles, CA 90095, United States of America; 2 Department of Environmental and Occupation Health, Joe C. Wen School of Population and Public Health, University of California Irvine, Irvine, CA 92697, United States of America

**Keywords:** pesticides, enforcement, methyl bromide, fumigation

## Abstract

Methyl bromide (MeBr) has been widely used as a fumigant to control for pests, fungi and weeds as well as for disinfection of warehouses, shipping containers, and other commodities. MeBr is a known developmental, neurologic and respiratory toxin. Due to its ozone-depleting properties, MeBr was listed under the Montreal Protocol in 1992. While MeBr use was set to phase out by 2005, the Montreal Protocol and the US Clean Air Act allows critical use exemptions, such as fumigation of freight containers for quarantine and preshipment purposes. To evaluate state-wide spatial and temporal patterns, we examine publicly available pesticide data on the use of MeBr in California from 2016–2022. We found that MeBr applications continue in 36 out of 58 CA counties. For non-agricultural fumigation applications (e.g., commodity fumigation) of MeBr from 2016–2022, a total of 582,050 kilograms (1,283,201 pounds) were applied across 25 counties in CA, home to 24 million people. Los Angeles ranks as the highest use county, with a total of 269,571 or 46% of the total kilograms (594,302 pounds) applied from 2016–2022 for non-agricultural fumigation applications. Additionally, we characterized ambient MeBr concentrations in the West Long Beach community of LA County, based on a state monitor active since 2023, observing concentrations exceeding CA standards. This study underscores the importance of evaluating chemical phaseouts and improving enforcement and monitoring to ensure public health protections.

## 1. Introduction

Methyl bromide (MeBr, bromomethane, CH3Br) is a halogenated aliphatic hydrocarbon and an effective broad-spectrum pesticide [1] Historically MeBr has been widely used for soil fumigation to control for pests, fungi and weeds as well as for disinfection of warehouses, buildings, shipping containers, wood and other commodities [2]. Due to its ozone-depleting properties, MeBr was listed under the Montreal Protocol in 1992 [3, 4]. With few exceptions, signatories, including the United States, agreed to the phase out of MeBr use and production by 2005 [5]. In 1994, the U.S. Clean Air Act further classified MeBr as an ozone depleting substance [6]. The actions taken by countries following the Montreal Protocol has resulted in over 85% of the total MeBr for all uses being phased out globally. However, on average, 10 million kilograms (10,000 tonnes) of MeBr continues to be used every year [7].

Methyl bromide is a known developmental, neurologic and respiratory toxin [8]. In 1993, MeBr was formally listed under California (CA) Proposition 65 as a developmental (reproductive) toxicant [9, 10]. Animal studies have consistently demonstrated that inhalation of MeBr is associated with respiratory issues and damage to the kidneys, skin and livers of mice and rats [11–14]. In workplace settings, MeBr exposures have been associated with various adverse health impacts including headache, eye irritation, weakness, dizziness, throat irritation, nausea, coughing, lightheadedness, itching, and runny noses with nasal irritation [15]. Chronic studies among occupationally exposed cohorts further suggest potential increased risk of stomach and prostate cancer [16–19] as well as chronic bronchitis [20]. Research among structural fumigation workers found adverse impacts on memory, cognitive function and neurobehavioral function [21, 22]. In children, associations have been found between MeBr and respiratory symptoms or use of asthma medication [23], asthma emergency department visits [24], lower birth weight and length and a smaller head circumference [10].

The Montreal Protocol and the US Clean Air Act allow for Critical Use Exemptions (CUEs) for continued MeBr use, despite the global phaseout [25]. Exemptions were granted until 2015 for strawberry growers in the state of CA and continued to be allowed in some cases for strawberry nurseries [5, 26]. After the near total ban of MeBr in CA agricultural fields, fumigation of freight containers with MeBr is still permitted, under quarantine and preshipment exemptions [27, 28]. Freight transported in containers is typically fumigated, as a means to reduce potential infestations or inhibit the spread of pests. In the Unites States (US), foreign freight containers with certain produce such as grapes, must be treated for pests, and in most cases, MeBr is allowed to be used to fumigate these containers at permitted facilities [29]. The US is the largest reported consumer of MeBr for quarantine and preshipment purposes, with a reported use of over 1.5 million kilograms (1500 tonnes) in 2023 [30]. We consider the case of MeBr use in CA, as it reported the highest agricultural use until 2015 and the state boast several large ports. Further, CA collected and publishes data on both agricultural and non-agricultural uses.

The adjacent ports of Los Angeles and Long Beach (known collectively as the San Pedro Bay Ports) handle approximately 40% of all sea container imports to the US, including imported fresh fruit [31]. Residents of neighborhoods near the Ports have expressed concern around exposures related to nearby fumigation facilities [32]. A state-run ambient air monitor for MeBr was established in west Long Beach in 2023. To understand more about spatial and temporal use patterns, we aimed to examine applications of methyl bromide by specific use categories (i.e., fruits nursey/greenhouse, soil/preplant, non-agricultural fumigation, vegetables and other) across the state from 2016–2022 and characterize ambient MeBr concentrations in the West Long Beach community based on a state monitor since 2023.

## 2. Methods

### 2.1. Study design, populations, and data sources

Data on MeBr applications from 2016–2022 across CA was obtained from the California Department of Pesticide Use Registry (CDPUR) [33]. CDPUR data has been previously described by other exposure and environmental epidemiological studies [10, 34]. Briefly, since 1990, CA requires mandatory reporting of all agricultural pesticide applications and in some cases non-agricultural applications (i.e., fumigation). Data is publicly available through the Pesticide Use Report (PUR) Program. The PUR data includes information on active ingredient, brand name, date/time of pesticide application, amount, and for agriculture usage acreage within 1-square mile sections (~1.6 km × 1.6 km) defined by the Public Land Survey System. Non-agricultural pesticide applications (e.g., for commodity fumigation) are only reported on a monthly basis at the county level. Information of daily applications for non-agricultural uses at a smaller geographical resolution are not readily available. Data on MeBr pesticide applications for 2023 has not been released through the CDPUR, but records for Los Angeles County were requested directly.

The PUR data undergoes extensive documentation, quality control and quality assurance through the CDPUR [35]. Briefly, the CDPUR implements the outlier program that involves utilizing a statistical method to detect possible errors in the database fields that document acres treated and the amount of pesticide used (in pounds). If errors are found, these are documented and provided as a separate .txt file. There are three different criteria to determine if a value is an outlier, these are 1) Pounds per acre of active ingredient is larger than 200 (for non-fumigants), or 1000 (for fumigants) 2) Pounds per unit treated of a product is larger than 50 times the median and 3) Pounds per unit of product is larger than a value generated using a neural network.

We abstracted information on facilities located in CA with MeBr permits from 2016–2023 from the California Emissions Inventory Data Analysis and Reporting System (CEIDARS) [36]. The CEIDARS database is compiled and maintained by the California Air Resources Board [37]. Data is collected from local air districts, state agencies and numerous other sources, and cross-referenced across other data. Each air district is provided with a comprehensive guide to conduct the emissions inventory. Validation of the data is automated with manual validation checks implemented throughout the process. The California Air Resources Board began conducting MeBr air monitoring in west Long Beach in 2023. Data on MeBr air concentrations were obtained from the CARB’s Methyl Bromide Data Dashboard [38]. Data on ambient MeBr air concentrations are not readily available across CA.

Information on the imports of grapes was obtained using the United States Department of Agriculture Agricultural Marketing Service MyMarketNews (MMN) Application Programming Interface (API) [39]. We queried the database specifically for data available on imports and shipments from the SC National Shipping Point Trends Report. The SC National Shipping Point Trends Report contained information on shipment units by week, commodity (e.g., apples, grapes), category (e.g., fruit, vegetables), and district. Data on a total of 51,140 records was only available beginning on February 15th, 2021. A total of 130 records were identified as grape imports through the Ports of Los Angeles/Long Beach. Lastly, we used the 2016–2020 American Community Survey to estimate the total population living in a county where any MeBr application occurred [40].

### 2.2. Data cleaning and quality assurance

Yearly data files on pesticide applications were downloaded from the CDPUR database directly. The files for the years 2016–2022 were merged together and filtered to only include ‘methyl bromide’ using the variable ‘chemname’. As CDPUR provides a separate .txt file with county names, the filtered file was merged with county identification codes. To ensure the data values were consistent with the CDPUR values, we compared the total amount of methyl bromide used by county from 2016–2022 with the annual CDPUR summaries. We found no discrepancies between our data summaries and CDPUR annual summaries for methyl bromide applied by county for years 2016–2022. Data are reported in pounds by the CDPUR, so we converted pounds to kilograms. As pesticide applicators are required to report any pesticide applications, any county with no data available is assumed to have a value of 0 pounds (kilograms) applied.

Yearly files on facilities located in CA with MeBr permits from 2016–2023 were downloaded directly from CEIDARS. All files were merged, summary on locations was conducted using the following variables provided: county where facility is located, air district responsible for location where facility is located, and city where facility is located.

### 2.3. Geospatial mapping

Geospatial maps summarizing the average kilograms (pounds) of methyl bromide across all categories and specific use categories (i.e., (i.e., fruits nursey/greenhouse, soil/preplant, non-agricultural fumigation, vegetables and other) by county were completed via R studio using the following libraries, tigris, sf, dplyr, ggplot2, and stringr. Using the tigris library we obtained California county boundaries for the year 2022. Prior to mapping, we projected all spatial data to the WGS84 coordinate reference system (EPSG:4326) using st_transform(4326) in R.

### 2.4. Statistical analyses

We categorized MeBr applications by the following application uses: fruits, vegetables, non-agricultural fumigation, soil fumigation, nursery/greenhouse, and other (e.g., landscape maintenance, structural pest control). Full site codes used for each categorization are provided in appendix (table S1). For each use category we summed the total kilograms of MeBr from 2016–2022 across all CA counties and provide trends and summaries on the total yearly average kilograms of MeBr applied. We provide a summary of the total monthly kilograms of MeBr applications for non-agricultural fumigation purposes from 2016–2023 in Los Angeles County. Additionally we summarized the average monthly MeBr ambient air concentrations by month and year in west Long Beach, CA from 2023–2025. Lastly, we conducted a spearman correlation to assess the relationship between monthly MeBr concentrations and monthly MeBr for non-agricultural fumigation applications that occurred in 2023 (monthly MeBr for non-agricultural fumigation applications in Los Angeles is only available through 2023). All statistical analysis were conducted via R Studio 4.5 (version 2025.05.0+496) [41].

## 3. Results

### 3.1. Descriptive analysis

**Figure 1.**
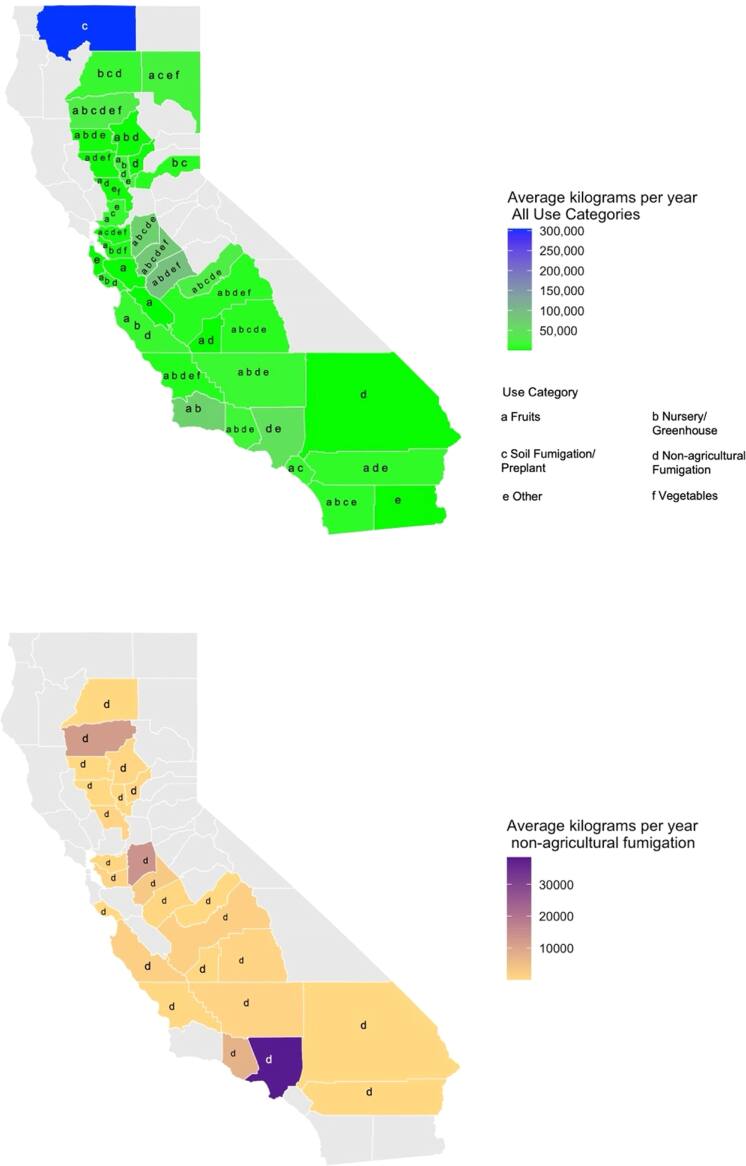
Yearly average kilograms of MeBr applications for all use categories (top) and non-agricultural fumigation purposes (bottom) by county across California from 2016–2022.

**Figure 2.**
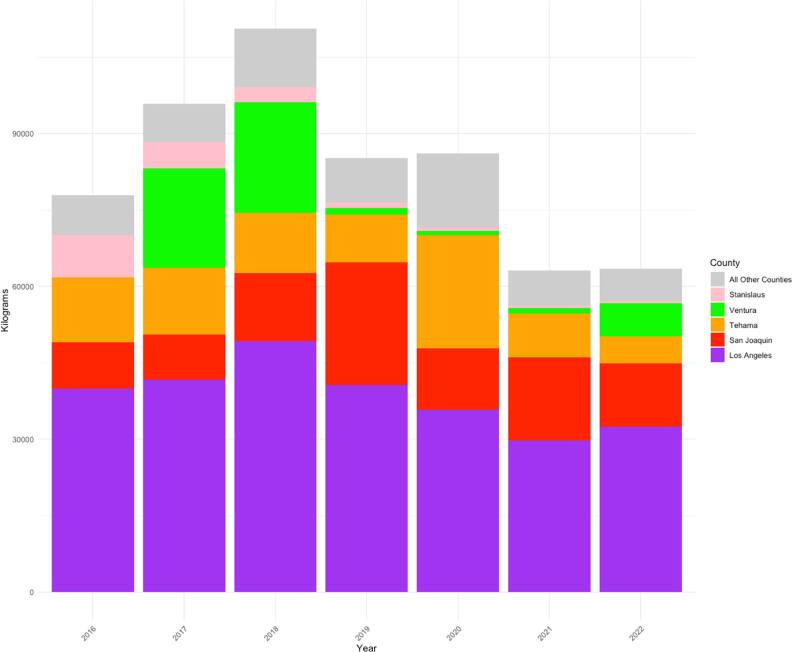
Top counties using methyl bromide (kilograms) for non-agricultural fumigation purposes by year (2016–2022).

Across the state of CA from 2016–2022, MeBr was used for 59 unique application types in 36 out of 58 counties (figure 1), home to over 35 million residents. The top counties in CA reporting MeBr applications across all uses (i.e., fruits, vegetables, non-agricultural fumigation, soil fumigation, nursery/greenhouse, and other) are Siskiyou, Merced, Stanislaus, San Joaquin County, and Los Angeles (Figure S1). Among the first four predominantly agricultural counties, the top uses for MeBr were soil fumigation (Siskiyou and San Joaquin), and nursery/greenhouse (Merced, San Joaquin, and Stanislaus). In Los Angeles, the primary use was non-agricultural fumigation (e.g., commodity fumigation) (Figure S1). Non-agricultural fumigation applications of MeBr from 2016–2022, totaled 582,050 kilograms (1,283,201 pounds). Application occurred in 25 counties in CA, home to over 24 million people (figure 1). Los Angeles ranks as the highest use county for non-agricultural fumigation, with a total of 269,571 or 46% of the total kilograms (594,302 pounds) applied from 2016–2022 in CA (figure 2, table S2). On average, since 2016, 38,510 kilograms (84,900 pounds) of MeBr is used in Los Angeles County for non-agricultural fumigation (figure 1, table S2). This is 2.8 times more than the next highest reported county (i.e., San Joaquin: 13,709 kilograms (30,223 pounds)) (table S2) and 5 times more than the next highest county with a seaport (Ventura County: 7,269 kilograms (16,026 pounds)) (table S2, figure S2).

### 3.2. Monthly trends in methyl bromide applications in Los Angeles County

**Figure 3.**
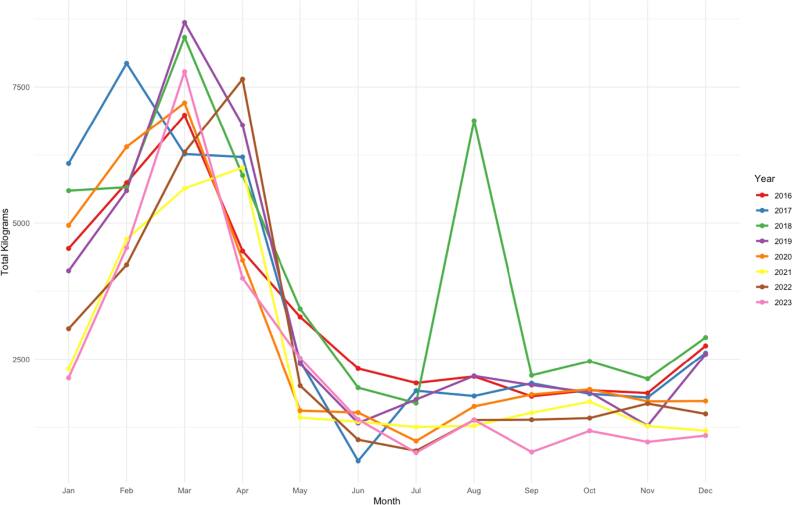
Total monthly kilograms of methyl bromide applications for non-agricultural fumigation purposes 2016–2023 in Los Angeles County, California.

The total monthly kilograms of MeBr applications from 2016–2023 for non-agricultural fumigation purposes in Los Angeles County ranges from 634 to 8411 kilograms (1397 to 18,543 pounds) (figure 3, table S3). The majority (60%) of reported MeBr use for non-agricultural fumigation occurs between January and April. Using the CEIDARS dataset we identified a total of 212 fumigation facilities having permits to emit MeBr across California from 2016–2023, with 181 having an active permit in 2023. There has been a total of 20 permitted fumigation facilities in LA County, with 19 fumigation facilities having an active permit as of 2023. The facilities in LA County are concentrated in densely populated areas of the Harbor Communities and Southeast LA cities, near the ports and freight transport corridors.

### 3.3. Ambient methyl bromide monitoring

**Figure 4.**
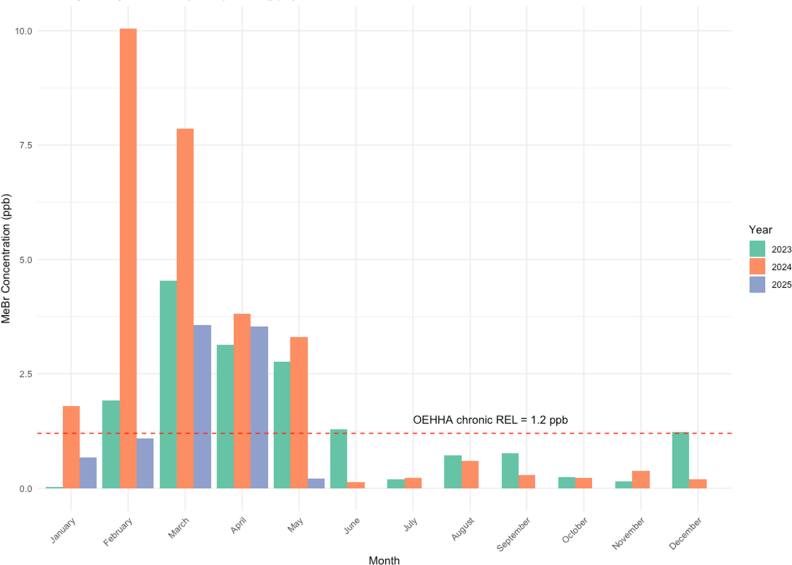
Average measured monthly ambient MeBr concentration (ppb) from 2023–2025 in Long Beach, California.

The highest measured ambient MeBr concentrations in west Long Beach for 2023, occurred during the months of February, March, and April (figure 4, table S4). In 2023, there is a correlation between monthly average MeBr applications and monthly MeBr average concentrations in the air (rho: 0.69, 95% CI (0.20–0.91), p-value:0.016). According to public officials MeBr is most commonly used in LA on the fumigation of imported grapes [32]. From February 2021 to July 2025 grapes imported into the San Pedro Bay ports occurred between the months of January-May (Figure S3), which coincide with the highest reported MeBr air concentrations in west Long Beach (figure 4). The majority of the grapes are reported as originating from the countries of Chile and Peru.

## 4. Discussion

Despite the global initiatives for a phaseout on the use of MeBr, with complete ban by 2015, we find that in CA MeBr continued to be used between 2016–2022 for agricultural pesticide applications in vegetables and fruits, soil fumigation, nursery/greenhouses, non-agricultural fumigation and other uses (e.g., outdoor plants or structures) (Figure S4). The use of MeBr in quarantine and preshipment of commodities continues to be exempted from the phaseout. However, the potential localized health burdens on the residents and workers due to continued use of MeBR are understudied. While MeBr is known as a developmental, neurologic and respiratory toxin, there is limited understanding of the health implications of fumigation of freight on nearby communities. Humans can be exposed to MeBr via inhalation and dermal contact. Studies among fumigation workers documented adverse health impacts in farmworker and freight workers including headache, eye irritation, weakness, dizziness, throat irritation, nausea, coughing, lightheadedness, itching, and runny noses with nasal irritation [15]. Research of structural fumigation workers found adverse impacts on memory, cognitive function and neurobehavioral function as well as cardiovascular health [21, 22, 42, 43]. To date, we are unaware of any epidemiological studies analyzing the potential community effects from the exposure to MeBr contaminants due to its use in shipping and storage [44].

The California Office of Environmental Health Hazard Assessment set non-cancer reference exposure level (REL) values for acute air exposure to MeBr at 3900 μg m^−3^ (neurologic targeted toxicity) and for chronic REL for the respiratory tract target to be 5 μg m^−3^ [45]. Data from the MeBr monitor in west Long Beach from January 2023 to April 2024 showed that, on average, *nearly double* the long-term health-protective reference exposure level — 2.1 ppb compared to 1.2 ppb (5 μg m^−3^) as measured by ambient community monitor (figure 4). These levels translate to a hazard quotient (HQ) of 1.6 (HQ = $\frac{{Air\, concentration}}{{REL}}=\frac{8.16\,{\mathrm{\mu }}{\mathrm{g}}/{\mathrm{m}}3}{5\,{\mathrm{\mu }}{\mathrm{g}}/{\mathrm{m}}3}$), indicating a potential for non-cancer health effects to increase. Additionally, on two occasions acute (hourly) levels of methyl bromide reached over 900 ppb [32]. The surrounding area is home to an elementary school, 600 homes, and nearby parks. This area has been state-designated as a disadvantage community (Figure S5) and is ranked in the top 25th percent of environmental hazards and social vulnerability [46].

**Children’s exposures require particular attention**. Methyl bromide , is a chemical that is heavier than air and therefore children’s exposure to this pesticide may be greater than that of adults [47]. Risks to children are uniformly higher than those of adults due to a greater inhalation rate-to-body weight ratio and other factors. Inhalation of fumigation pesticides in children is an exposure pathway that is rarely investigated [48]. Research in rural agricultural communities have seen an association between proximity to agriculturally applied MeBr and restricted fetal growth [10], but not childhood cancer [49]. Furthermore, increase in the ambient air concentration of MeBr is associated with increased risk of pediatric asthma emergency department visits for 6 to 18 year olds in California [24].

Data from the CADPR has been used to provide annual summaries on pesticide use patterns. While it relies on self-reported data from pesticide applications, it has been used for research studies [10, 34, 50]. However, unlike agricultural pesticide applications that are available on a daily level, and at the following geospatial resolutions: ***CO**unty, **M**eridian, **T**ownship, **R**ange, and **S**ection (*COMTRS*),* pesticide applications classified as non-agricultural pesticide applications (e.g., commodity fumigation) are only required to be reported at the monthly and county level. This presents a limitation as information on exposure to non-agricultural pesticide applications cannot be tied to an individual facility to best characterize susceptible neighborhoods. Additionally, the data only being available at a monthly and county level likely attenuates short-term exposure peaks and spatial heterogeneity [51]. As the PUR data relies on self-reported data, it is prone to underestimation or misclassification bias due to systematic errors. It is possible that those completing the reporting forms may report usage of MeBr using wrong/inconsistent site codes (table S1). In addition to ensuring the accuracy of the reports through audits, efforts should be made to improve reporting requirements of non-agricultural fumigation applications. Furthermore, as the only state in the US that makes pesticide use data publicly accessible, it is difficult to determine which areas nationwide are the highest consumers. Annual reports by the California Department of Pesticide Regulation provide information on all pesticides applied by county, while this information is valuable, information on specific use categories (e.g., amount applied on fruits or nurseries) are not readily summarized.

Another limitation in this study pertains to the MeBr air concentrations. Only monthly MeBr air concentrations from a single CARB monitor located near two-fumigation facilities in Long Beach is available beginning in 2023 [32]. Despite these limitations we were able to determine that Los Angeles County is the top County in California using MeBr for non-agricultural fumigation. Only nine additional non-agricultural fumigants are reported to be used in Los Angeles County (e.g., Sulfuryl Fluoride, Aluminum Phosphide, Magnesium Phosphide, etc) (Figure S6). Taking these additional non-agricultural fumigants into account, MeBr is still the top non-agricultural fumigant being used in Los Angeles County. Furthermore, data on permits for fumigation facilities from CEIDARS and data on grape imports from USDA can also be prone to administrative errors.

## 5. Conclusion

This analysis identified continued use of methyl bromide in communities across the state of CA. Since the phase out of MeBr began there have been various alternatives investigated as possible replacements [52]. Integrative pesticide management approaches are one way to reduce the reliance on chemicals. These include, the use of non-chemical methods that have the potential to address pests such as treatment with inert gases or cold treatments [53]. Fumigant alternatives, such as ethyl formate, have demonstrated efficacy at addressing pest and are considered to be less harmful for humans and the environment [53–56]. It is important to ensure that any chemicals being considered as alternatives undergo appropriate evaluations. For example, while, sulfuryl fluoride, emerged as a potential alterative to MeBr [57], it was not without risk, as impacts to the central nervous system have been observed in fumigation workers [21]. Lastly, while there is a requirement that MeBr capture technology be used on fumigation facilities to decrease offsite migration of the gas, it is unclear how often these are inspected [58, 59]. The inspection of equipment is critical, as leaks in fumigation can lead to human exposure as well as a decrease in effective fumigation [60].

This work highlights the ongoing challenge of addressing community health concerns due to fumigation activities and the lack of comprehensive health and safety assessments. The potential health burdens for continued MeBr use are concentrated in areas near fumigation activities, however there are remaining gaps in information and oversight. There are a multitude of agencies at the local, state and federal levels with a role in approvals and oversight of MeBr use (e.g., County agricultural commissioner, CA EPA, U.S EPA, US Department of Agriculture), however, there is also a lack of coordination and communication across the agencies. Cooperation and interagency engagement could provide a more comprehensive understanding of the potential public health impacts and implementation of effective approaches to enforcement of MeBr use. A more robust notification system, similar to that for field pesticide use, would offer alerts to residents or schools regarding fumigation activities. Currently, only pesticides used for agricultural purposes are included in CalEnviroscreen 4.0 [61]. Excluding non-agricultural pesticide applications from tools such as such as CalEnviroscreen 4.0 underestimates the cumulative burden faced by these communities [62, 63].

Future work should focus on conducting MeBr air monitoring near facilities where non-agricultural fumigation is occurring. Additionally, as there are minimal studies in non-occupational settings, efforts should be made to focus on understanding the potential health effects from exposure to MeBr. This study highlights the continued use of MeBr in densely populated communities and with close proximity to sensitive receptors.

## Data Availability

The data that support the findings of this study are openly available at the following URL/DOI: https://files.cdpr.ca.gov/pub/outgoing/pur_archives/; https://ww2.arb.ca.gov/capp/cst/ch2/wcwlb/methyl-bromide.
